# Loss of Stim2 in zebrafish induces glaucoma-like phenotype

**DOI:** 10.1038/s41598-024-74909-0

**Published:** 2024-10-18

**Authors:** Sofiia Baranykova, Rishikesh Kumar Gupta, Arkadiusz Kajdasz, Iga Wasilewska, Matylda Macias, Aleksandra Szybinska, Tomasz Węgierski, Karim Abu Nahia, Shamba S. Mondal, Cecilia L. Winata, Jacek Kuźnicki, Lukasz Majewski

**Affiliations:** 1 Laboratory of Neurodegeneration, International Institute of Molecular and Cell Biology in Warsaw, Ks. Trojdena 4, 02-109 Warsaw, Poland; 2 Institute of Bioorganic Chemistry, Polish Academy of Sciences, Zygmunta Noskowskiego 12/14, 61-704 Poznan, Poland; 3 Xenstats sp. z o.o., Otwarta 1, 60-008 Poznan, Poland; 4 Mossakowski Medical Research Institute, Polish Academy of Sciences, Adolfa Pawińskiego 5, 02-106 Warsaw, Poland; 5 Microscopy and Cytometry Facility, International Institute of Molecular and Cell Biology, Ks. Trojdena 4, 02-109 WarsawWarsaw, Poland; 6 Laboratory of Zebrafish Developmental Genomics, International Institute of Molecular and Cell Biology in Warsaw, Ks. Trojdena 4, 02-109 Warsaw, Poland; 7 Present Address: Amity Institute of Neuropsychology & Neurosciences, Amity University, Noida, 201313 India

**Keywords:** Stim2, SOCE, Retina, scRNA-seq, GABAergic amacrine cells, Glaucoma, Mechanisms of disease, Retina, Gene expression

## Abstract

Calcium is involved in vision processes in the retina and implicated in various pathologies, including glaucoma. Rod cells rely on store-operated calcium entry (SOCE) to safeguard against the prolonged lowering of intracellular calcium ion concentrations. Zebrafish that lacked the endoplasmic reticulum Ca^2+^ sensor Stim2 (*stim2* knockout [KO]) exhibited impaired vision and lower light perception-related gene expression. We sought to understand mechanisms that are responsible for vision impairment in *stim2* KO zebrafish. The single-cell RNA (scRNA) sequencing of neuronal cells from brains of 5 days postfertilization larvae distinguished 27 cell clusters, 10 of which exhibited distinct gene expression patterns, including amacrine and γ-aminobutyric acid (GABA)ergic retinal interneurons and GABAergic optic tectum cells. Five clusters exhibited significant changes in cell proportions between *stim2* KO and controls, including GABAergic diencephalon and optic tectum cells. Transmission electron microscopy of *stim2* KO zebrafish revealed decreases in width of the inner plexiform layer, ganglion cells, and their dendrites numbers (a hallmark of glaucoma). GABAergic neuron densities in the inner nuclear layer, including amacrine cells, as well as photoreceptors significantly decreased in *stim2* KO zebrafish. Our study suggests a novel role for Stim2 in the regulation of neuronal *insulin* expression and GABAergic-dependent vision causing glaucoma-like retinal pathology.

## Introduction

Calcium signaling is implicated in several processes in the retina and underlies various eye pathologies. The dysregulation of calcium homeostasis and calcium signaling is associated with neurodegenerative conditions (reviewed in^1–4^). Glaucoma is one such pathology, characterized by damage to the optic nerve, a tract of retinal ganglion cell axons that are covered with myelin^5^. In glaucoma, retinal ganglion cells (RGCs) undergo numerous alterations, including interruption of the retrograde axonal transport of neurotrophic factors, oxidative and endoplasmic reticulum (ER) stress, and intracellular Ca^2+^ overload^6^. This results in a progressive and irreversible decrease in the number of RGCs^3,7^. The specific mechanism of calcium-dependent glaucoma has not been established. Calpains are calcium-dependent proteases that have been implicated in calcium-dependent glaucoma^8–12^, in addition to several other causes, such as glutamate receptor overexcitation, high extracellular glutamate levels, changes in the activity of voltage-operated calcium channels, the activation of transient receptor potential 4 (TRPV4), reverse activity of the Na^+^/Ca^2+^ exchanger, the activity of protein kinase C (PKc), and mitogen-activated protein kinases (MAPKs)^1,4,13–16^. Glutamate excitotoxicity in glaucoma has been linked to an increase in Ca^2+^ influx into mitochondria^17–19^. In all of these pathways, the pathologies were associated with an increase in Ca^2+^ levels. However, in rod cells, a low level of Ca^2+^, which occurs under sustained saturating light conditions, is neurotoxic. To prevent cytosolic levels of Ca^2+^ from exhaustion, rods use store-operated calcium entry (SOCE)^20^.

SOCE is the mechanism by which the ER is refilled with Ca^2+^ and involved in Ca^2+^ signaling^21–23^. It is activated by ER Ca^2+^ sensors, called stromal interaction molecules (STIMs), which induce the opening of plasma membrane Ca^2+^ channels, such as calcium release-activated calcium channel protein Orai and transient receptor potential channels (TRPCs). We reported the presence of *STIM* and *Orai* transcripts in cortical neuron cultures of the rat brain^24^ and subsequently provided evidence of the interaction of endogenous STIM and Orai in neurons and their involvement in SOCE^25^. The STIM family includes STIM1 and STIM2 proteins, which have different functions and sensitivities to Ca^2+^ level in the ER^26^. It is noteworthy that Ca^2+^ ions that enter cells via SOCE not only replenish intercellular stores but also participate in cellular signaling, for instance, affecting the activity of transcription factors^21^. High level of STIM2 protein was observed in the central nervous system of rodents and zebrafish, and STIM2 was shown to be crucial for SOCE in mouse neurons. Furthermore, *Stim2* KO mice exhibit alterations of the vision system^27^, including cataracts^28^, but no changes in retina were reported^27^. In lenses with cataracts, an increase in intracellular Ca^2+^ levels was observed, and SOCE was detected in the lens of epithelial cells and retinal pigment epithelial (RPE) cells^29–31^. Although STIM2 has been linked to neurodegeneration^32^, it remains unknown whether it can be also relevant in the pathogenesis of glaucoma.

Zebrafish (*Danio rerio*) possess all necessary genes for SOCE, including duplicated *stim1* (*stim1a* and *stim1b*) and duplicated *stim2* (*stim2a* and *stim2b*)^33^. The knockout of either *stim2a*^34^ or *stim2b*^35^ resulted in increased locomotor activity and anxiety-related thigmotaxis in zebrafish larvae. These larvae exhibited moderate abnormalities in phototactic response, yet their visual-motor response remained normal, indicating that their vision was not affected. However, experiments with *stim2a*;*stim2b*^-/-^ double KO (hereinafter referred to as *stim2* KO) revealed that Stim2 deficiency in zebrafish resulted in vision impairment, as evidenced by the loss of phototactic behavior, abnormal visual-motor response, alterations of the stratum pigmentosum, as well as thinning of the inner plexiform layer (IPL) and RGCs^36^. Furthermore, alterations in gene expression were observed in the *stim2* KO larvae, including a reduction in the expression of genes involved in light perception and an increase in the expression of *anxa3a*, a marker of activated microglia. Importantly, the thinning of RGCs and the IPL, changes in microglial function, and impaired vision in *stim2* KO zebrafish resemble pathologies in glaucoma^37–39^.

The present study sought to understand the mechanisms that are responsible for vision problems that are caused by the loss of Stim2. The objective of this study was to ascertain whether Stim2 deficiency affects the gene expression profile in the retina or in a particular subset of neuronal cells, and whether the observed effects are associated with the vision deficits detected in *stim2* KO larvae. We used bulk RNA sequencing (RNA-seq), single-cell RNA (scRNA)-seq, immunohistochemistry, confocal microscopy, and transmission electron microscopy to identify differences between *stim2* KO zebrafish and a line that expressed both forms of Stim2 (referred to as control). We used zebrafish lines with genetically encoded fluorescent probe under a neuronal promoter to investigate the impact of *stim2* KO in fluorescence-activated cell sorting (FACS)-purified neurons by scRNA-seq. We observed *insulin* among differentially expressed genes (DEGs) in specific neuronal cell clusters, which might underlie STIM2 associated pathologies at the cellular level. We found that features that are characteristic of glaucoma, such as reduction in IPL width and the number of ganglion cells and their dendrites, were also observed in *stim2* KO zebrafish. Moreover, the analysis of γ-aminobutyric acid (GABA)-positive cells in the inner nuclear layer (INL) revealed their reduction, suggesting the importance of Stim2 in GABA-dependent neuronal properties.

The present study has demonstrated that Stim2 plays a pivotal role in the regulation of neuronal *insulin* expression, as well as in the maintenance of proper retinal morphology and function. Altogether, our results suggest that zebrafish depleted of Stim2 is a suitable model to investigate glaucoma-related changes.

## Results

### Bulk transcriptomics analyses in* stim2* KO zebrafish

**Fig. 1:**
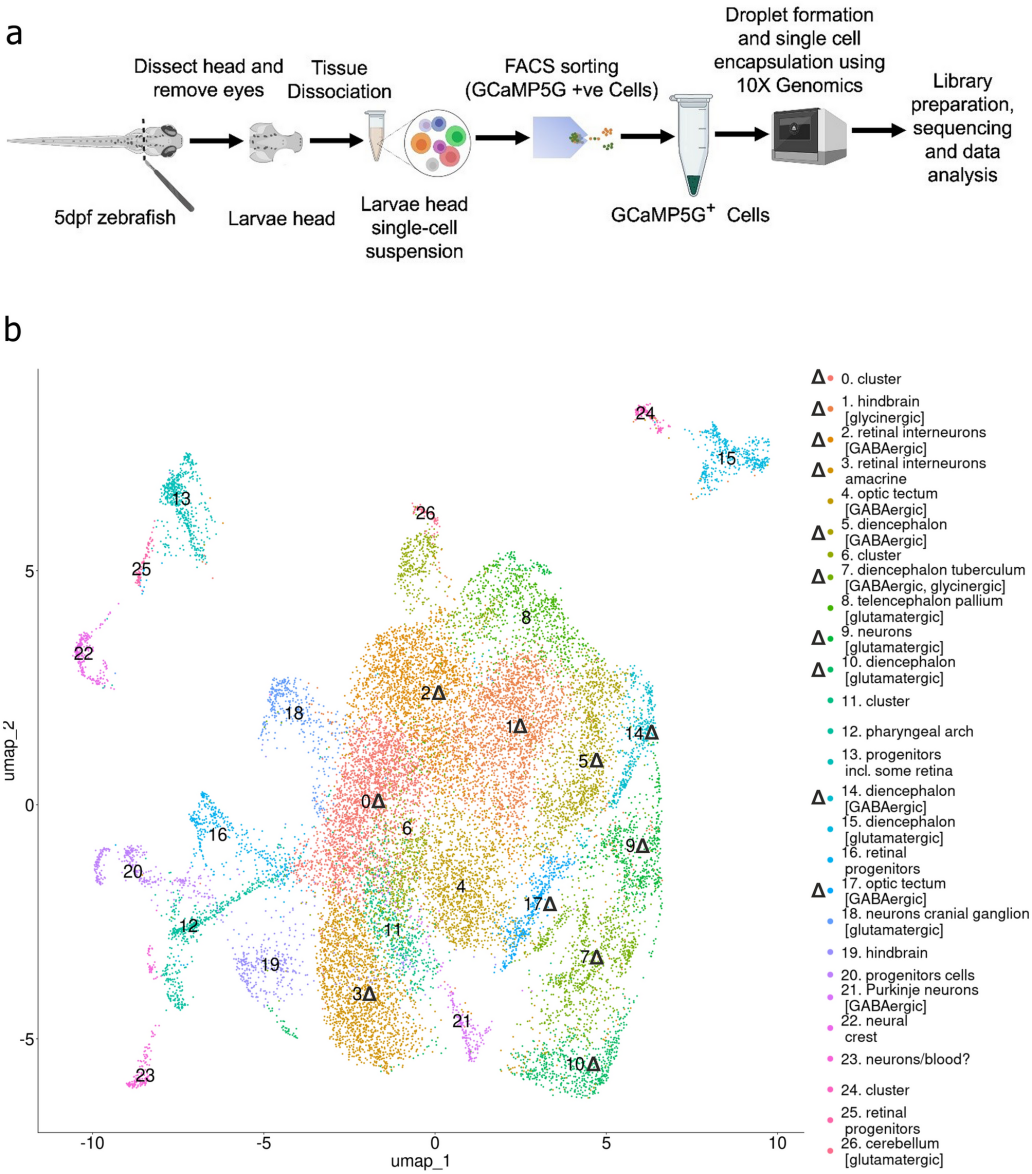
Single-cell RNA-seq analysis of cells of neuronal origin. (**a**) Schematic of cell preparation and processing of the 5 dpf zebrafish brain. The procedure begins with the dissecting of the zebrafish larvae head and removal of the eyes. The larvae heads are then dissociated to create a single-cell suspension. Fluorescence-activated cell sorting (FACS) is used to isolate GCaMP5G-positive cells, which are indicative of neuronal origin. These cells are encapsulated in droplets using 10× Genomics technology for single-cell RNA sequencing (scRNA-seq). The encapsulated cells undergo library preparation, sequencing, and subsequent data analysis to assess gene expression. (**b**) UMAP representation demonstrates the distribution of control and *stim2* KO zebrafish brain cells of neuronal origin. A total of 27 clusters were identified. Upright triangle symbols (Δ) show clusters with DEGs between *stim2* KO and control cells (adjusted *p* < 0.05). Thirty larvae were used for each sample, and two samples of each line *stim2* KO and controls were sequenced.

In samples from whole *stim2* KO zebrafish larvae, vast changes in gene expression were observed^36^. However, it remained unknown whether Stim2 is also important in transcriptional regulation in the retina. To characterize changes in retinal gene expression patterns that are induced by the loss of Stim2, we performed bulk RNA-seq in micro-dissected eyes from 5-days postfertilization (dpf) *stim2* KO larvae. We identified 32520 genes that were expressed across all samples, which was reduced to 22145 after filtering for low read count. Correlation analyses suggested no significant differences between genotypes (Fig. 1Sa). The top 20 most highly expressed genes, based on log-normalized count data, are shown in Fig. 1Sb and contained several marker genes for eyes (*opn1-sw1* and *cgrygm2d10,* encoding opsin 1 and crystallin, respectively), confirming eye-origin of the analyzed samples. Differential gene expression analyses (p_adj_ [*p*-values adjusted] < 0.05) revealed four downregulated genes and one gene showing a tendency towards upregulation (p_adj_ = 0.06) (Table 1). However, the functions of protein products of those genes have not been elucidated and are based only on predictions, which makes it challenging to relate them with previously observed problems with vision and alterations of the stratum pigmentosum^36^.

**Table 1:** List of differentially expressed genes (DEGs) identified by comparing *stim2* KO and control zebrafish eyes by bulk RNA-seq.

Genome resources	log2FoldChange	padj	External_gene_name
**ENSDARG00000114577**	**− 1.44031**	**0.00022**	**si:dkey-159n16.2**
**ENSDARG00000017474**	**− 1.53886**	**0.00074**	**zgc:110699**
**ENSDARG00000013771**	**− 2.57138**	**0.04227**	**ctss2.2**
**ENSDARG00000095409**	**− 3.70662**	**0.04659**	**si:ch211-226h7.8**
ENSDARG00000074345	3.38432	0.05957	si:ch211-214b16.4
ENSDARG00000102593	6.91001	0.06087	si:ch211-214b16.2
ENSDARG00000069464	− 2.23002	0.09196	cox7a1
ENSDARG00000074150	− 3.09644	0.09196	si:ch211-226h7.5
ENSDARG00000105489	− 2.33418	0.13919	loc564660
ENSDARG00000109267	− 4.77828	0.15421	CABZ01024501.1
ENSDARG00000075600	− 1.07494	0.17401	si:dkeyp-41f9.3
ENSDARG00000097746	− 2.01457	0.17401	si:rp71-77l1.1
ENSDARG00000100106	− 3.50763	0.17401	CR385054.1
ENSDARG00000074919	0.78166	0.18413	BFSP1
ENSDARG00000070480	− 2.21236	0.18413	agr2
ENSDARG00000104919	− 6.35518	0.18413	si:ch211-153b23.3
ENSDARG00000105183	1.68275	0.22626	CT009487.2
ENSDARG00000045142	− 4.28075	0.22626	hbae5

The low number of identified DEGs indicates no major difference between both samples. Genes with *p*_adj_ < 0.05 are shaded in bold.

Among the downregulated genes were *si:dkey-159n16.2* (which encodes a protein that is predicted to have adenosine triphosphate [ATP] binding activity and involved in intracellular signal transduction), *zgc:110699* (which is predicted to have guanosine triphosphate [GTP] binding activity and GTPase activity and involved in signal transduction), *si:ch211-226h7.8* (which is predicted to be involved in neutrophil migration and active in the apical plasma membrane, cell surface, and extracellular space [orthologous to human glycoprotein]), and *tss2.2* (which is predicted to have cysteine-type endopeptidase activity, involved in the immune response and proteolysis, and localized to the extracellular space and lysosome). The human ortholog of this gene (*CTSS*) encodes cathepsin S and is implicated in neuropathy. It is expressed in blood, macrophages, and the yolk syncytial layer. The only gene that was upregulated was *si:ch211-214b16.4*, which encodes a protein that is predicted to have ATP binding activity and involved in intracellular signal transduction.

In summary, the bulk RNA-seq analysis of the eye did not identify genes that could possibly explain vision impairment in *stim2* KO zebrafish. However, the earlier bulk RNA-seq of whole *stim2* KO larvae identified downregulated genes that are related to light perception^36^. This prompted us to search for genes in separated neuronal cells using scRNA-seq in the samples extracted from the brain of larval zebrafish rather than the eyes.

### Graph-based clustering of single-cell transcriptome analysis reveals distinct neuronal cell populations

**Fig. 2:**
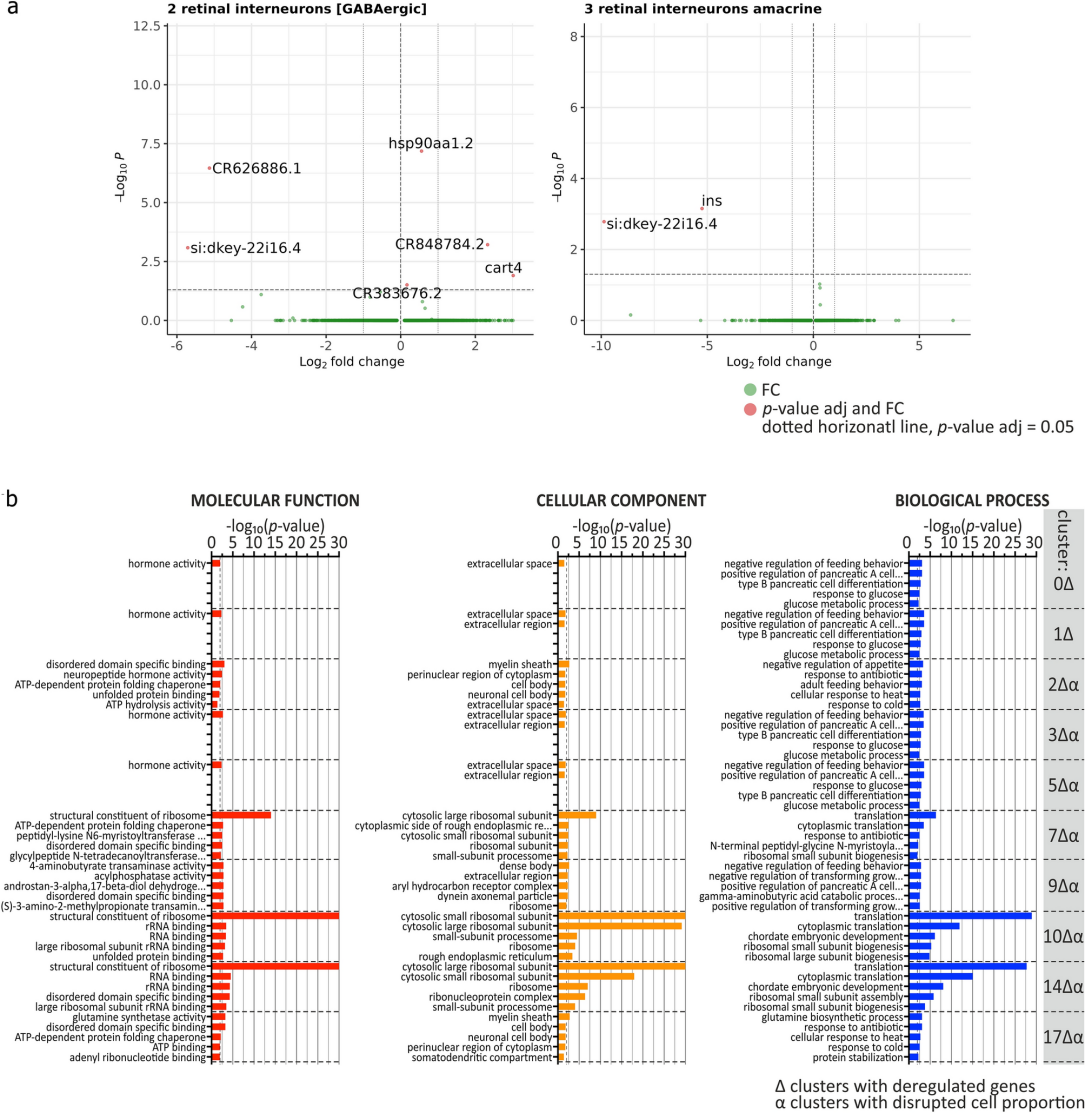
Differentially expressed genes in different clusters in *stim2* KO zebrafish and the GO analysis. (**a**) Volcano plots of differentially expressed genes in clusters 2^Δ^ and 3^Δ^. Genes significantly deregulated in each cluster are labeled. Horizontal dotted line indicates *p*-value adjusted = 0.05; green dot represent genes with not-significantly changed expression; red dots represent genes with significantly changed expression (*p*-value adjusted < 0.05 and log_2_ fold change (FC) different from 0). (**b**) Gene Ontology enrichment analysis of DEGs showing molecular function (red bars), cellular component (orange bars), and biological process (blue bars) aspects of 10 clusters with DEGs. All GO terms are presented for *p* < 0.05 (Fisher’s test). Up to five GO terms are presented for each cluster. ^Δ^, clusters with dysregulated genes identified; ^α^, clusters with dysregulated proportion of cells.

**Fig. 3:**
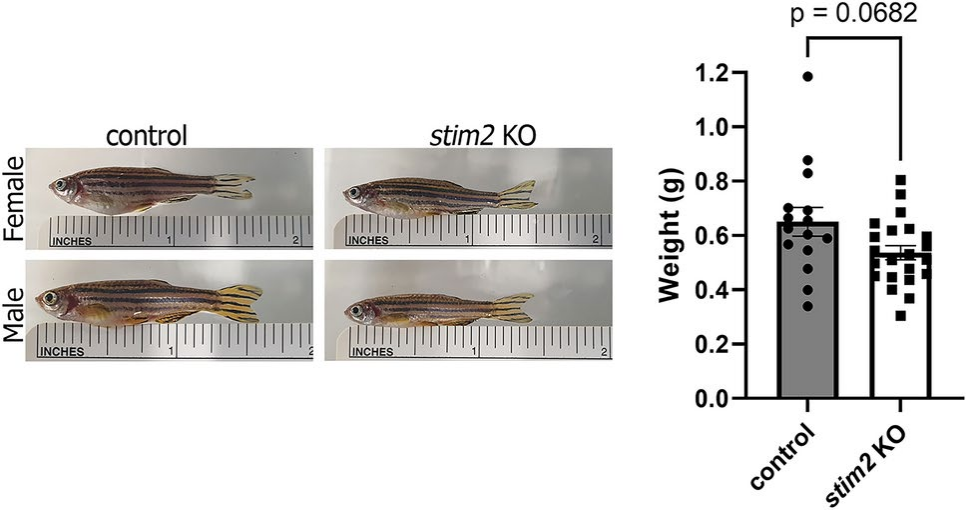
Changes in weight between *stim2* KO zebrafish and control adult fish. *stim2* KO zebrafish weighed considerably less than controls. *n* = 22 *stim2* KO (7 females, 15 males), 15 control (7 females, 8 males). The scatter dot plots represent mean values ± standard error of the mean. Statistical analyses were performed using an unpaired *t*-test with Welch’s correction. Data passed the Shapiro–Wilk normality test and Rout method for eliminating outliers.

Single-cell RNA-seq was performed on brain cells of neuronal origin of 5 dpf zebrafish larvae that expressed green fluorescent probe under the *huc*/*elavl3* promoter^40^ (Figs. 1a, 2S). Single-cell suspensions were obtained by the enzymatic and mechanical digestion^41^ of heads without eyes from two fish lines: *stim2* KO and control. After obtaining the single-cell suspension, the cells were sorted by fluorescence-activated cell sorting (FACS) based on their green fluorescence (Fig. 3S).

Twenty-seven distinct cell clusters were identified and visualized using the Uniform Manifold Approximation and Projection (UMAP) approach (Fig. 1b). Based on known marker genes that were provided by the Daniocell database^42^, we assigned cell types to clusters. Twenty-three clusters were defined in this way, but remaining four were devoid of any known cell types. The top 10 gene markers for each cluster are shown in Table 1S.

### Differentially expressed genes in clusters of *stim2* KO zebrafish are associated with the GABA-ergic neurons

**Fig. 4:**
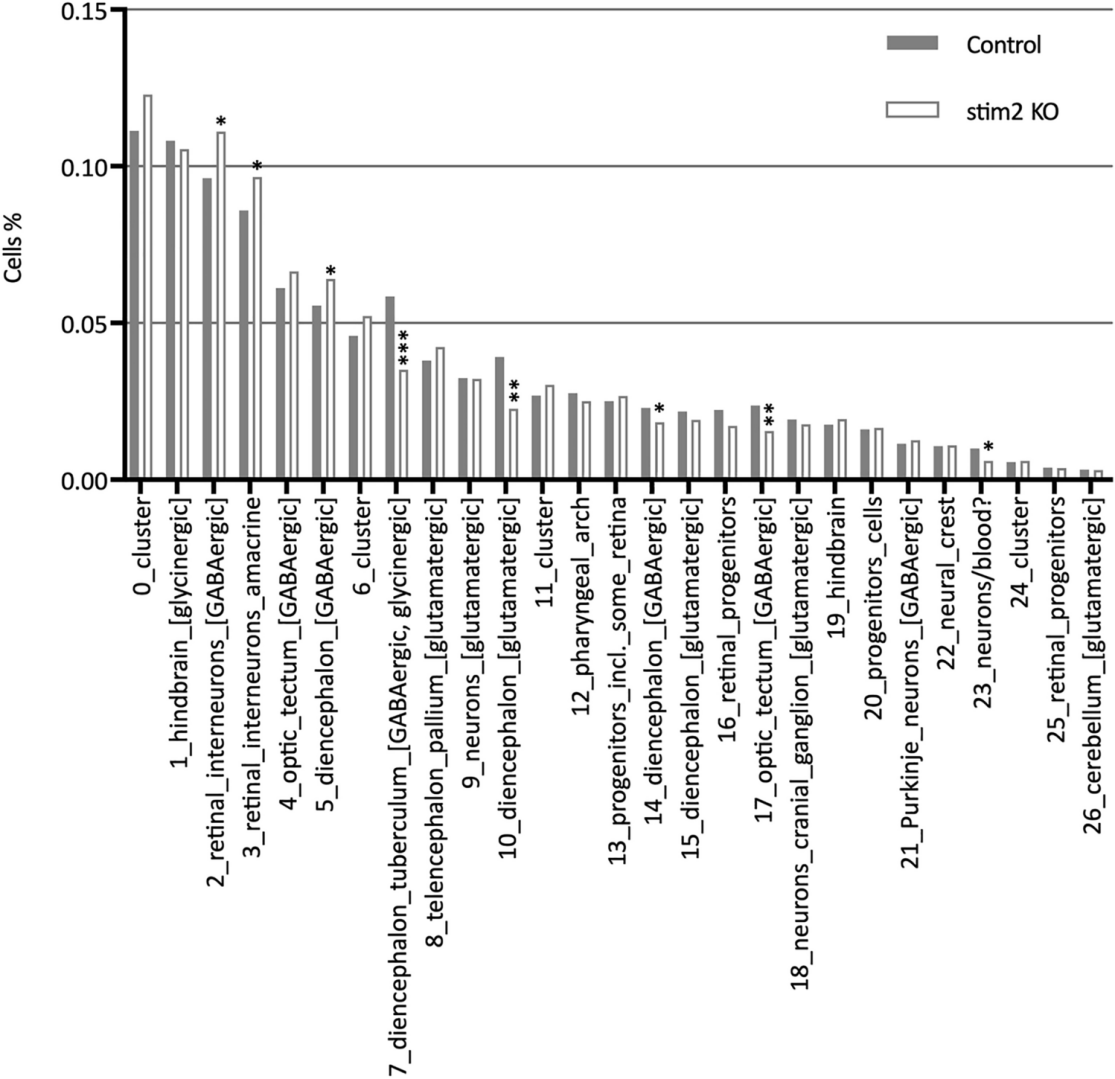
Cell proportions in *stim2* KO zebrafish (white bars) and control (gray bars) clusters. Asterisks mark clusters with significant changes in cell proportions in *stim2* KO relative to control. Significance of change in cells proportions in clusters were calculated using *t*-test with Benjamini and Hochberg FDRs (False Discover Rates, *FDR < 0.05, **FDR < 0.01, ***FDR < 0.001). The proportion of cells was calculated using the propeller function (speckle v. 1.2 package) with arcsin square root transformation (see “Materials and methods”).

Differentially expressed genes in clusters across conditions were identified with the nonparametric Wilcoxon rank sum test. Differentially expressed genes with adjusted *p*-values below 0.05 and a minimum two-fold change were found in 10 clusters. Interestingly, more than half (6/10) of the identified clusters with DEGs contain GABAergic neurons, including GABAergic retinal interneurons (cluster 2^Δ^) and amacrine retinal interneurons (cluster 3^Δ^; Table 2; Fig. 2a shows volcano plots for these two clusters). In GABAergic retinal interneurons (cluster 2^Δ^), two genes were upregulated: *cart4* (eight-fold) and *CR848784.2* (five-fold). First one encodes cocaine- and amphetamine-regulated transcript 4, the latter is unannotated. In the same cluster, two genes were downregulated: *si:dkey-22i16.4* (52-fold) and *CR626886.1* (35-fold). In amacrine retinal interneurons (cluster 3^Δ^), there were two downregulated genes: *ins* (38-fold) and *si:dkey-22i16.4* (935-fold). Overall, *ins*, which encodes insulin, was downregulated at least 38-fold in six clusters, and *si:dkey-22i16.4*, which encodes a secretory calcium-binding phosphoprotein, was downregulated at least 20-fold in four clusters (Table 2, Fig. 4S). *CR848784.2* was upregulated in three clusters at least five-fold, and *acyp1* was upregulated in two clusters at least three-fold (Table 2). The latter encodes a small cytosolic enzyme that catalyzes hydrolysis of the carboxyl-phosphate bond of acylphosphates and is widely expressed in tissues, including the retina.

**Table 2:** List of clusters with DEGs between *stim2* KO and control including GO analysis.

Name and cluster number DEGs (down and up)	GO top biological processes based on DEGs	Change in number (#) of cells between *stim2* KO vs control
0^Δ^-unidentified Down: *ins*, *si:dkey-22i16.4*	Negative regulation of feeding behavior Positive regulation of pancreatic A cell differentiation	–
1^Δ^-hindbrain [glycinergic] Down: *ins*, *si:dkey-22i16.4*	Negative regulation of feeding behavior Positive regulation of pancreatic A cell differentiation	–
2^Δ^-retinal interneurons_GABAergic Down: *CR626886.1*, *si:dkey-22i16.4* Up: *CR848784.2*, *cart4*	Negative regulation of appetite Response to antibiotic	# cells 15% up
3^Δ^-retinal interneurons_amacrine Down: *ins*, *si:dkey-22i16.4*	Negative regulation of feeding behavior Positive regulation of pancreatic A cell differentiation	# cells 12% up
5^Δ^-diencephalon [GABAergic] Down: *ins*, *si:dkey-22i16.4*	Negative regulation of feeding behavior Positive regulation of pancreatic A cell differentiation	# cells 15% up
7^Δ^-diencephalon_tuberculum [GABAergic, glycinergic] Down: *nucb2a*,* rps17* Up: *grin1b*, *oaz2b*,* CR7883163*	Translation Cytoplasmic translation	# cells 40% down
9^Δ^-neurons_[glutamatergic] Down: *rps17*, *nocta*, *si:ch211-181d7.1.1*,* ins* Up: *CR848784.2*, *acyp1*, *hsd20b2*, *selenoj*	Negative regulation of feeding behavior Negative regulation of transforming growth factor β activation	–
10^Δ^-diencephalon_[glutamatergic] Down: *mgst3b10*, *txnl4a10*,* rps17* Up: *hsp90aa1.2*,* mknk2b10*	Translation Cytoplasmic translation	# cells 42% down
14^Δ^-diencephalon [GABAergic] It is similar to cluster 5 but exhibits DEG Down: *si;dkey42i9.4*, *calr3b*,* rps17* Up: *CR848784.2*, *camk2b1*, *abat, acyp1*	Translation Cytoplasmic translation	# cells 20% down
17^Δ^-optic tectum [GABAergic] Up: *glula*, *ppdpfb* , *hsp90aa1.2*	Glutamine biosynthetic process Response to antibiotic	# cells 34% down

Genes that exhibited at least 1.5 fold change in the expression level relative to control are presented.

To ascribe functional relevance to the DEGs, we performed GO enrichment analysis in the clusters that are shown in Table 2 in all three GO classes: biological process, cellular component, and molecular function (Fig. 2b). In most clusters with DEGs, negative regulation of feeding behavior (clusters 0^Δ^, 1^Δ^, 2^Δ^, 3^Δ^, 5^Δ^, 9^Δ^), positive regulation of pancreatic A cell differentiation (clusters 0^Δ^, 1^Δ^, 3^Δ^, 5^Δ^), and translation and cytoplasmic translation (clusters 7^Δ^, 10^Δ^, 14^Δ^) were affected (Fig. 2b). Interestingly, the dysregulated expression of genes in 5 dpf larvae that are related to the regulation of feeding behavior was accompanied by a substantial tendency toward a smaller weight in adult *stim2* KO zebrafish (Fig. 3). Additionally, the observed changes in cell proportions in specific clusters, such as the increase in GABAergic retinal interneurons and amacrine retinal interneurons, underscore the potential impact of Stim2 on neuronal cell type composition and function.

### Changes in the proportion of GABAergic type neurons between *stim2* KO zebrafish and control

Proportions of cells were significantly affected (False Discovery Rate [FDR] < 0.05) in eight clusters. Except cluster 23, all others belonged to clusters with DEGs (Table 2, Fig. 4). Therefore, the majority of affected cell clusters were those containing GABAergic cells. In three clusters, the proportion of cells from *stim2* KO larvae was elevated (by 15% in cluster 2^Δ^, by 12% in cluster 3^Δ^, and by 15% in cluster 5^Δ^; Table 2, Fig. 4). In five clusters from *stim2* KO zebrafish, the number of cells decreased compared with control larvae (by 40% in cluster 7^Δ^, by 42% in cluster 10^Δ^, by 20% in cluster 14^Δ^, by 34% in cluster 17^Δ^, and by 38% in cluster 23 [neurons/blood?]). This analysis demonstrated that the loss of Stim2 not only affects gene expression in GABAergic neurons, but also has an impact on the number of those cells in the zebrafish larvae brain. The changes in cell proportions, particularly the increased number of GABAergic retinal interneurons and amacrine retinal interneurons in *stim2* KO zebrafish, reflect a possible compensatory mechanism or a disruption in neuronal development and differentiation. This shift in cellular composition may contribute to altered synaptic connectivity and neurotransmission, potentially leading to functional impairments.

### Detection of GABA-positive cells and photoreceptors in *stim2* KO and control zebrafish

**Fig. 5:**
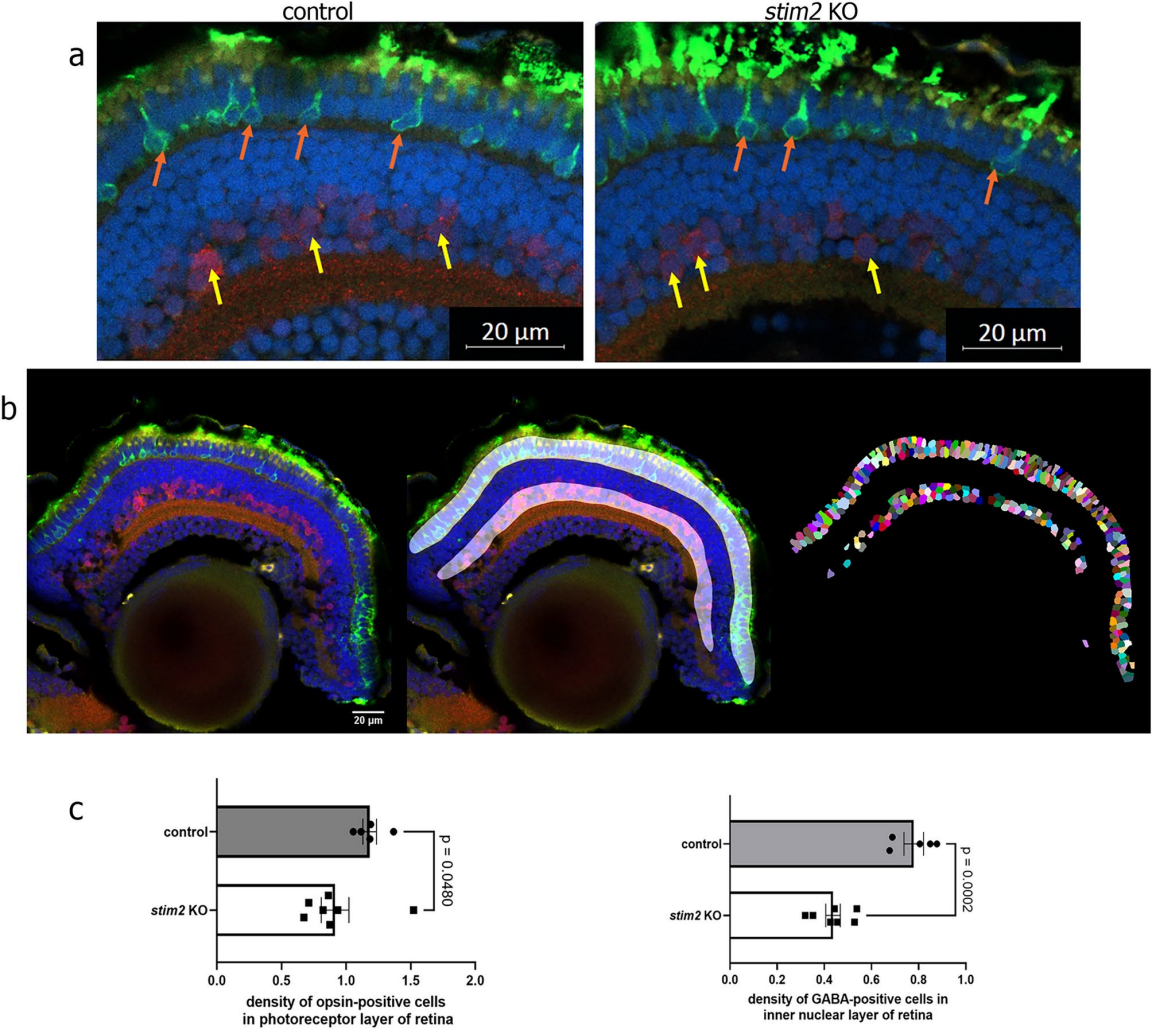
Immunofluorescence visualization of cells in the retina of 5 dpf zebrafish larvae. (**a**) Photoreceptors (orange arrows) were stained with anti-opsin antibodies (green), and amacrine cells (yellow arrows) were stained with GABA antibodies (red). Nuclei were stained with Hoechst 33342 (blue). **(b**) (left) Representative retina image stained as above. (middle) Masks covering the inner nuclear (INL) and photoreceptor layers (PL) (right). Labeled nuclei contained in those layers. (**c**) Cell densities among the investigated retina layers, estimated as the number of cells that were positive for opsin or GABA above the fluorescence and size thresholds and normalized to surface volume of the mask. The photoreceptor layer and INL were 1.29 × and 1.78 × larger in control retinas of 5 dpf larvae. *n* = 7 *stim2* KO, 5 control. The scatter dot plots represent mean values ± standard error of the mean. In the case of the photoreceptor cell density analysis, the Mann–Whitney test was conducted because the data did not pass the Shapiro–Wilk normality test. Statistical analyses of INL cell density were performed using an unpaired *t*-test with Welch’s correction because the data passed the Shapiro–Wilk normality test. The Rout method for eliminating outliers did not reveal any outliers among the tested conditions.

Single-cell RNA sequencing of samples extracted from the brains of larval zebrafish revealed an increased proportion of cells expressing GABAergic amacrine interneuron markers in *stim2* KO versus control (Table 2, Fig. 4). This is in contrast to previously observed thinner ganglion cell layer (GCL), which contains GABAergic amacrine neurons^43^, in the retina of adult *stim2* KO zebrafish^36^. To elucidate the apparent discrepancy between these observations, we conducted an analysis of the number of amacrine cells in the GCL and inner nuclear layer (INL) of the larval retina. To identify amacrine cells that are present in the GCL and INL, anti-GABA antibodies were used. Additionally, to stain photoreceptors, anti-opsin antibodies were used (Fig. 5).

**Fig. 6:**
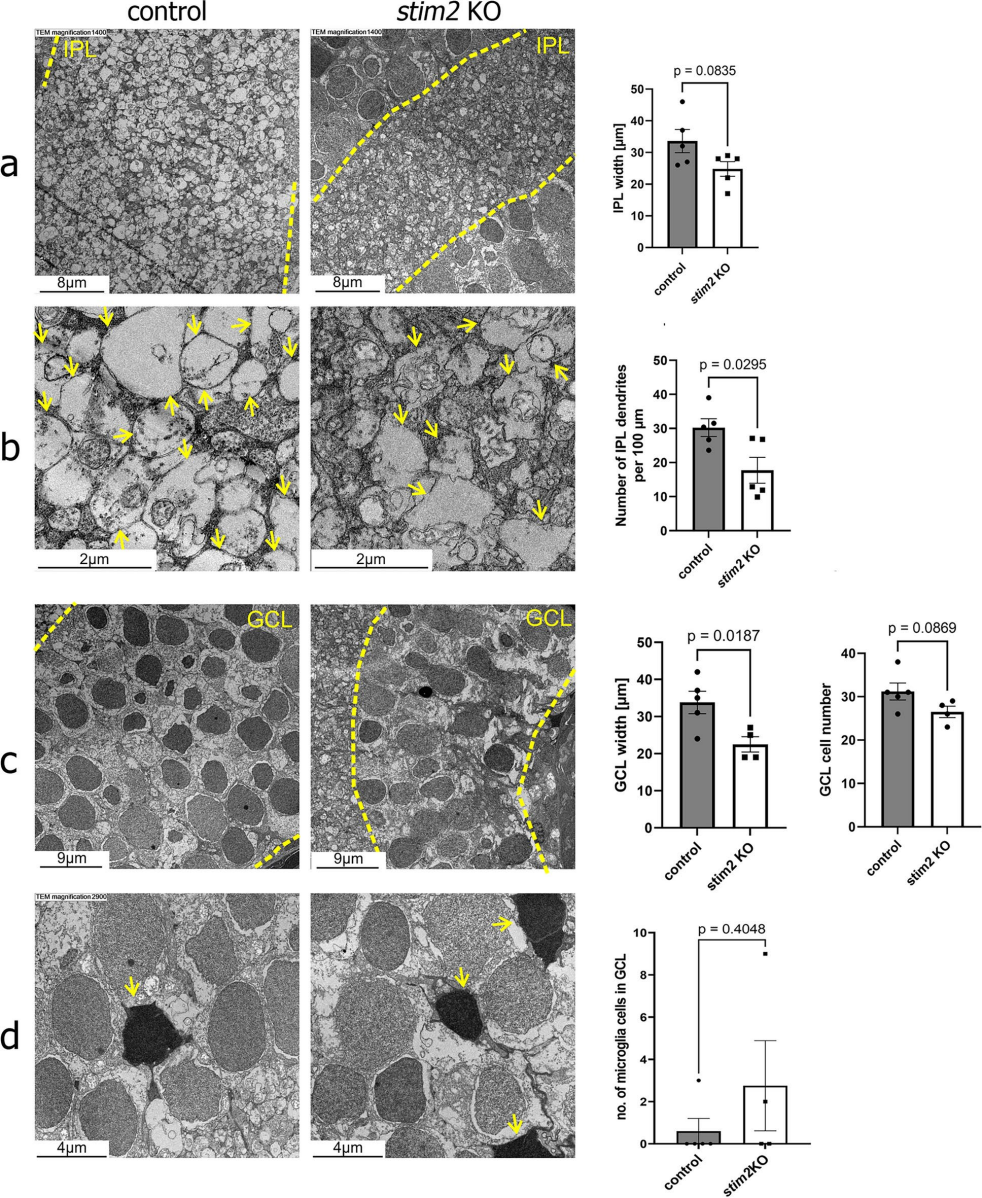
Transmission electron microscopy analysis revealed a decrease in IPL width as a consequence of ganglion cell perturbations. Left images show control samples and right images—*stim2* KO samples. (**a**) Narrowing of the IPL in *stim2* KO retina with visible malformations of ganglion cell dendrites. The yellow dotted lines indicate borders of the IPL. (**b**) The number of dendrites decreased in the IPL in *stim2* KO zebrafish, and their shape was altered. Arrows point to dendrites. (**c**) Narrowing of the GCL in *stim2* KO zebrafish, with a substantial decrease in the number of ganglion cells. Yellow dotted lines indicate borders of the GCL. The scatter dot plots represent mean values ± standard error of the mean. Images from at least four larvae were analyzed in (**a**–**c**). Statistical analyses were performed using an unpaired *t*-test with Welch’s correction. Data passed the Shapiro–Wilk normality test. (**d**) The number of microglia within GCL increased (arrows indicate the microglia cells). The Mann–Whitney test was conducted because the data did not pass the Shapiro–Wilk normality test; at least 5 larvae per variant were analyzed.

The analysis of cell density in the INL, which consists of horizontal cell bodies, bipolar cells, and amacrine cells, showed a significant decrease (1.78 × lower) in the number of GABAergic neurons in the retina in *stim2* KO, suggesting that Stim2 may be involved in the regulation of GABAergic synaptic transmission, which plays an important role in shaping the retinal response to visual stimuli^44^ (Fig. 5c). Quantitative analysis of the photoreceptor layer in *stim2* KO zebrafish revealed a significant reduction in the density of opsin-positive cells, specifically those expressing rhodopsin, indicating a decrease in the rod photoreceptor population (Fig. 6c). Both results suggest that Stim2 plays an important role in rod-dependent vision, such as dark and light adaptation^45^, and explain our previously demonstrated impairment in light preference^36^.

### Transmission electron microscopy analysis reveal malformation in the *stim2* KO retina structure

To comprehensively assess the impact of Stim2 deficiency on retinal ultrastructure, we conducted a thorough transmission electron microscopy (TEM) analysis across all retinal layers, with particular focus on the inner plexiform layer (IPL), ganglion cell layer (GCL), and photoreceptor layer. This systematic examination allowed us to identify and characterize structural alterations associated with the *stim2* KO. In 5 dpf *stim2* KO larvae, the GCL, INL, and IPL (also the inner synaptic layer) were narrowed compared with controls^36^. The IPL contains synapses between axons of bipolar cells and dendrites of ganglion cells. A decrease in the width of the IPL (Fig. 6a, c) and a reduction of the number of dendrites, suggesting their retraction, were also observed (Fig. 6a, arrows, 6c). Moreover, TEM analysis revealed that the number of ganglion cells was lower in *stim2* KO zebrafish (Fig. 6b, c). Additionally a substantial increase in the number of microglia cells was evident (Fig. 6d), which is consistent with our previously published data suggesting their increased activation^36^. Altogether, our results indicate for the first time that lack of Stim2 might be involved in shaping GABAergic connectivity in the retina likely by the modulation of microglia activation.

**Fig. 7:**
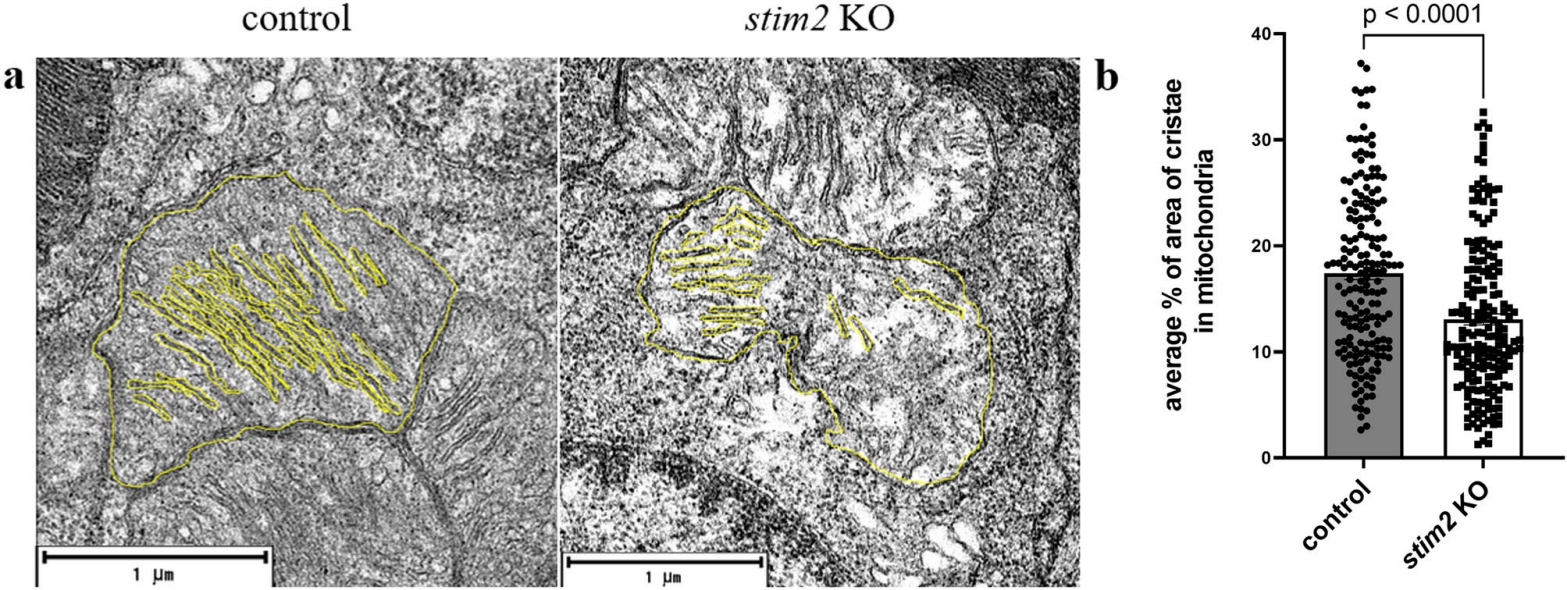
Cristae area in mitochondria of zebrafish photoreceptors. (**a**) Transmission electron microscopy image of mitochondria from 5 dpf zebrafish larvae with hand-labeled cristae. (**b**) Ratio of cristae area to area of mitochondria measured using ImageJ software in three pairs of control and *stim2* KO zebrafish. Each pair contained at least 40 TEM images of 190 *stim2* KO mitochondria and 160 controls. The data were analyzed using unpaired *t*-test.

Further quantitative analysis of photoreceptor mitochondria ultrastructure revealed significant alterations in *stim2* KO zebrafish (Fig. 7). Specifically, the cristae area in photoreceptor cells of *stim2* KO zebrafish was markedly reduced compared to control retinas (Fig. 7). This reduction in cristae area suggests a potential impact of Stim2 deficiency on mitochondrial morphology. Given the critical role of mitochondrial cristae in energy production, these structural changes may have profound implications for energy metabolism in retinal photoreceptors. This finding provides insight into the potential mechanisms by which Stim2 deficiency could affect photoreceptor function and survival, potentially contributing to the observed visual deficits in our model.

## Discussion

Glaucoma comprises a group of neurodegenerative diseases of optic nerve that can be caused by different factors and has a complex etiology. During glaucoma, RGCs and their axons undergo numerous alterations, including interruption of the retrograde axonal transport of neurotrophic factors, oxidative stress, ER stress, *N*-methyl-D-aspartate (NMDA) receptor activation, and intracellular Ca^2+^ overload^6^. Damage to RGC axons results in their progressive neurodegeneration and vision loss^3^. Axonal degeneration triggers rapid alterations of RGC dendritic arbors, leading to synaptic rearrangements (reviewed by^46^). Retinal ganglion cell dendrites receive synaptic inputs from bipolar and amacrine cells, allowing the flow of visual information. In glaucoma, the function of RGCs may be compromised due to alterations in their dendritic connections with bipolar cells within the IPL.

We propose that the *stim2* KO zebrafish might be useful for studying some features of glaucoma, such as the thinning of RGCs and the IPL, which we observed in *stim2* KO larvae^36^ and occur in glaucoma^37–39^. As *Stim2* KO mice exhibited pronounced cognitive deficits and survived only a few weeks after birth^47,48^, therefore investigating long-term effects of *Stim2* KO in rodents is not feasible, making zebrafish an attractive alternative. We generated zebrafish KO of *stim2a*, *stim2b*, and both, which were viable^34,35^. The lack of both Stim2 isoforms affected vision-related behaviors^36^ and retinal structure in *stim2* KO zebrafish. The phototaxis of *stim2* KO larvae was found to be disrupted, with the majority of the examined fish exhibiting no light preference or even a reversal of preference in comparison to wild-type fish^36^. A comparable, though less pronounced, phenomenon was observed in the case of single *stim2a* and *stim2b* KO^34,35^. Additionally, the visual-motor response of *stim2* KO larvae was abnormal, as they did not increase their mobility upon switching the light off^36^, in contrast to wild-type zebrafish and single *stim2a* and *stim2b* KO^34,35^. These findings suggest that both zebrafish Stim2 isoforms may be involved in visual perception and that the effects of their depletion are cumulative. Moreover, *stim2* KO larvae and adults possessed a thicker RPE^36^, which plays a key role in maintaining the photoreceptor layer^49^. Histological and TEM analyses further demonstrate the structural and cellular alterations in the retina of *stim2* KO zebrafish. The reduced number of GABAergic neurons and photoreceptors, along with the increased microglial activation, indicate that Stim2 plays a crucial role in maintaining retinal integrity and function. Our previous work demonstrated that Stim2 deficiency leads to increased *anxa3* expression, a marker of microglial activation^30^. This finding is particularly intriguing given the crucial role of microglia in maintaining neural homeostasis through their dynamic functions, including migration, phagocytosis, and cytokine production. Previous research has established a link between store-operated calcium entry (SOCE) and microglial properties, particularly in regulating phagocytosis and cytokine secretion^50^. The dendritic synapse loss observed in our study may be attributed to the disruption of Ca^2^⁺ homeostasis in microglia and their subsequent interactions with GABAergic neurons. Our current TEM data, showing an increased number of microglia in the ganglion cell layer (GCL), corroborates our earlier findings^36^.

STIM2 plays a significant role in regulating gene expression in neurons through several mechanisms. One of these is its involvement in SOCE, which can activate transcription factors such as CREB, NFAT and NFκB and thus modulate gene expression^51^. Another is the maintenance of basal calcium levels in neurons, which is crucial for normal neuronal excitability^52,53^. Although there is currently no direct evidence linking STIM1/2 with glaucoma, TRPC1 and TRPC6 channels, which are known interacting partners of STIM isoforms, were upregulated in glaucomatous lamina cribrosa leading to NFAT4-mediated gene expression remodeling^54^.

In both *stim2a* and *stim2b* single knockout zebrafish the changes in gene expression have been observed^34,35^. However, the analysis were conducted on samples from whole larvae. Consequently, it remains unclear whether the regulation of gene expression by Stim2 is dependent on the neuronal type, brain region or physiological context. This makes it challenging to identify DEGs specific to particular cell populations. To address this issue, we employed bulk RNA-seq analysis of the eye and scRNA-seq in neurons to examine alterations in gene expression in diverse neuronal cells following Stim2 depletion.

The bulk RNA-seq of eye samples revealed only a limited number of differentially expressed genes, which initially posed challenges in correlating molecular changes with the observed phenotype. This discrepancy might be attributed to the limitations of bulk analysis, where subtle alterations in specific cell subsets could be obscured by the overall tissue composition.

Interestingly, previously performed bulk RNA-seq analysis of whole larvae yielded more pronounced results. We observed downregulation of key adhesion molecules crucial for retinal structure maintenance, including *pcdh15b*, *prph2b*, *rom1a*, and *rom1b*. Additionally, the eye development transcription factor *crx* showed reduced expression. Notably, we detected a significant decrease in various vision-related genes, encompassing opsins, rhodopsin kinase (*grk1a*), phosphodiesterase (*pde6ha*), cGMP-gated cation channels, and metabotropic glutamate receptor 6 (*grm6b*)^36^.

To delve deeper into the neuronal impacts of Stim2 loss, we conducted single-cell RNA-seq on dissected zebrafish brains. This approach allowed us to examine gene expression profiles in distinct neuronal subpopulations. The contrast between the modest changes observed in eye samples and the more pronounced differences in whole larvae and brain samples is particularly striking. This disparity suggests that Stim2 may exert a stronger influence on gene expression in brain neurons compared to ocular tissues. In 10 out of 27 identified clusters of cells of neuronal origin from brains of 5 dpf *stim2* KO zebrafish statistically significant differences in gene expression in comparison to control were detected. In three clusters (amacrine retinal interneurons, GABAergic retinal interneurons and GABAergic diencephalon cells), the proportion of cells increased in *stim2* KO. Thus, there is an apparent discrepancy between the increased proportion of cells expressing GABAergic amacrine interneuron markers in the brain and reduced numbers of GABAergic amacrine cells in the retina of stim2 KO zebrafish. This modest yet statistically significant increase in the population of these cells may be indicative of a compensatory effect in response to the reduction in GABAergic amacrine cells in the retina. Additionally, it is important to consider that the observed alterations in the proportion of GABAergic cells in different regions of the brain may reflect aberrant development of these cells due to dysregulated gene expression. It is well-established that Ca^2+^ signaling plays a crucial role in the maturation and migration of GABAergic interneurons, suggesting that the absence of Stim2 could potentially impact this process^55,56^. Moreover, in five clusters with DEGs, the proportion of cells in *stim2* KO zebrafish versus control larvae significantly decreased, including GABAergic diencephalon and GABAergic optic tectum cells. A disrupted balance between inhibitory and excitatory signaling in the optic tectum affects neuronal activity and may be linked with our previous observations of a higher frequency of Ca^2+^ oscillations in optic tectum neurons in single *stim2a*^34^ and *stim2b*^35^ KO lines.

In clusters with higher or unchanged numbers of cells (0^Δ^, 1^Δ^, 2^Δ^, 3^Δ^, 5^Δ^), the negative regulation of feeding behavior and, surprisingly, the positive regulation of pancreatic A cell differentiation were observed. The effect on feeding correlates well with the observed loss of weight in *stim2* KO zebrafish and was likely attributable to downregulation of the *ins* (which encodes insulin). Moreover, it correlates with the lower deposition of glycogen in the liver in adult *stim2* KO zebrafish^36^. These findings suggest a systemic impact of loss of Stim2 on metabolic regulation and energy homeostasis. Whether Stim2 participates in insulin metabolism in neurons has not yet been established, but our work indicates its role in the neuronal expression of *ins* gene as well as of *si:dkey-22i16.4*.

The *si:dkey-22i16.4*, a gene encoding a secretory calcium-binding phosphoprotein, has not been previously linked to vision. This protein is involved in calcification processes, such as tooth formation in medaka and zebrafish^57,58^. The *si:dkey-22i16.4* is expressed in various tissues in cyprinid fish, including the eye and brain^59^. Whether and how the decrease in expression of *si:dkey-22i16.4* in retinal cell clusters in *stim2* KO zebrafish is linked to the observed glaucoma-like retinal pathology are still unclear. However, the fact that both the *si:dkey-22i16.4* and *ins* genes are highly downregulated in the same cell clusters is unlikely accidental. Notably, the calcification of trabecular meshwork was observed in open-angle glaucoma^60^. Aortic calcifications and dental dysplasia were described in Singleton-Merten syndrome, a rare immunogenetic disorder that features juvenile open-angle glaucoma^61,62^. Calcification of the carotid artery into the optic canal may play a role in the pathogenesis of low-tension glaucoma, called soft glaucoma^63^ (reviewed by^64^). It can thus be postulated that the disruption of calcification associated with the downregulation of *si:dkey-22i16.4* may underlie the glaucoma-like features observed in *stim2* KO zebrafish.

Insulin may be implicated in the pathology of vision. Insulin promotes substantial dendrite and possibly synapse regeneration, mediated by mammalian/mechanistic target of rapamycin complex 1 (mTORC1) and mTORC2^65^. Several studies demonstrated the potential of modulating insulin signaling in the treatment of neurodegenerative diseases, including glaucoma^66^. Diabetic patients have a higher risk of glaucoma^67–70^, whereby cataracts are formed earlier and progress faster^71^. Thus, our observation that *ins* expression significantly decreased in selected cell clusters, such as GABAergic retinal interneurons and amacrine retinal interneurons, supports links between glucose levels and eye pathology.

In this study, we present novel findings on the relationship between Stim2 and neuronal insulin expression. While previous research has established links between SOCE and insulin secretion in pancreatic β cells^72^, our data implies a unique role for Stim2 in neuronal *ins* gene transcription. We demonstrated that Stim2 deficiency leads to significantly reduced *ins* expression in several identified neuronal clusters (Fig. 4S), that include in the majority GABAergic neurons. This observation aligns with and extends prior work by Molnár et al.^73^, which identified GABAergic neurogliaform cells as local sources of insulin in the cerebral cortex. STIM2 role in the regulation of neuronal *ins* expression has not been previously reported. Given the critical role of insulin in neuronal survival, plasticity, and cognitive function^74^, these findings open an unexplored area for investigation of the interplay between calcium signaling, insulin expression, and GABAergic neurons function. Furthermore, these results provide a potential mechanistic link to studies demonstrating the effects of intranasal insulin on brain function, suggesting a complex regulatory network involving SOCE, Stim2, and neuronal insulin production^75^.This work provide a basis for further investigation into the tissue-specific regulation of *ins* expression and its implications for neurological function and disorders.

**Fig. 8:**
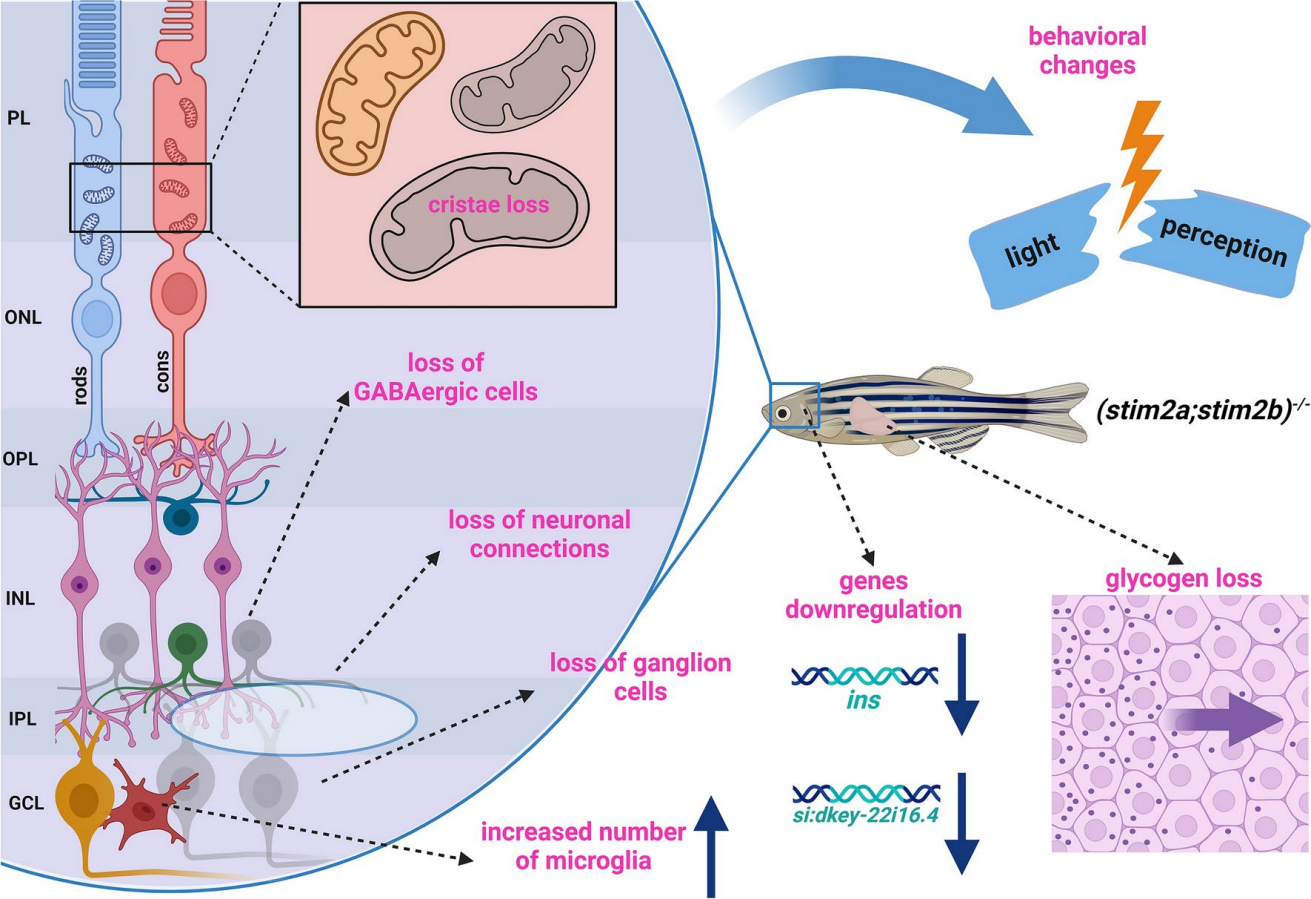
Pleiotrophic effect of *stim2* knockout resembling glaucoma like phenotype in zebrafish. Loss of Stim2 leads to impairment of GABAergic connections, resulting in loss of ganglion cells, causing changes in light perception. Increase in microglia number and their activation^36^, glycogen deficiency^36^ as well as mitochondria alterations may be a consequence of the insulin downregulation seen in *stim2* KO. GCL, Ganglion cell layer; IPL, inner plexiform layer; INL, inner nuclear layer; OPL, outer plexiform layer; PL, photoreceptor layer.

Retinal ganglion cells contain the highest number of mitochondria compared with other retinal cells to fulfill the higher energy requirements of efficient action potential propagation involved in highly dynamic visual-motor communication^70^. In glaucoma, RGC mitochondria undergo oxidative stress, leading to greater susceptibility to degeneration^70^. Similarly, photoreceptors also rely heavily on mitochondrial function for their high energy demands and calcium homeostasis^76^. In *stim2* KO zebrafish, we observed significant loss of cristae in mitochondria of photoreceptors (Fig. 8), suggesting that STIM2 plays a crucial role in maintaining mitochondrial integrity in these cells. This observation aligns with previous studies demonstrating the importance of calcium signaling in photoreceptor function and survival^77^. However, the limited scope of this study does not allow for the identification of the exact mechanism of the observed changes. It is possible that loss of Stim2 disrupts mitochondrial Ca^2+^ homeostasis, which may result in cristae disorganization and an impairment of the energetic balance of retinal cells.

In conclusion, our study elucidates the multifaceted role of Stim2 in neuronal gene regulation and cellular physiology, offering insights into its involvement in shaping neuronal connectivity and potential implications for glaucoma pathophysiology. We observed significant structural and functional alterations in the retina *stim2* KO zebrafish, including dendrite retraction in the inner plexiform layer (IPL), decreased dendrite numbers, substantial reduction in the retinal ganglion cell (RGC) layer. Furthermore, this study reveals a significant decrease in GABAergic neurons and a considerably smaller population of rhodopsin-positive rod cells in *stim2* KO zebrafish. These results implicate Stim2 in the regulation of GABAergic synaptic transmission (Fig. 8), which is crucial for shaping retinal responses to visual stimuli^44^ and rod-dependent vision, including dark and light adaptation^45^. This observation aligns with our previous demonstration of light preference loss in *stim2* KO larvae^36^. The specific changes in gene expression observed in neuronal cell clusters point to alterations in the insulin-mediated signaling (Fig. 8). These findings provide a basis for further investigation into the tissue-specific regulation of insulin expression and its implications for neurological function and disorders. The complex interplay between Stim2, calcium homeostasis, and insulin signaling in the context of microglial-GABAergic neuron communication presents a promising area for future investigation.

However, our observation that lack of Stim2 in zebrafish induces changes in retina similar to those observed in glaucoma have some limitations. The specific pathway responsible for the observed pathologies remains unknown, and it has not be determined if both of the genes that were found to be highly downregulated participate in this process. Future studies will address these issues and help to establish the suitability of *stim2* KO zebrafish as a model of glaucoma-like pathology taking into account that any single model perfectly replicates human glaucoma.

## Materials and methods

### Statement of ethics

All experiments were approved by Advisory Team for Animal Welfare Committee of International Institute of Molecular and Cell Biology in Warsaw and conducted in accordance with the European Communities Council Directive (63/2010/EEC) as described in detail previously^36^. All zebrafish were maintained according to internal regulations in the Zebrafish Core Facility (PL14656251, registry of the District Veterinary Inspectorate in Warsaw; 064 and 051, registry of the Ministry of Science and Higher Education) at the International Institute of Molecular and Cell Biology (IIMCB) in Warsaw (IN-MOL-CELL infrastructure). This study is reported in accordance with ARRIVE guidelines^78^.

### Animal husbandry

The Tg(*HuC:GCaMP5G*) zebrafish line^79^, AB zebrafish line, and *stim2* KO line that expressed GCaMP5G under the same promoter were used. The animals were maintained in the Zebrafish Core Facility under standard conditions^80^.

### Bulk RNA-seq

The procedure for bulk RNA-seq analysis was conducted according to our previous study^36^. Total RNA was isolated from zebrafish eyes. To synthesize sequencing libraries, a two-step approach was applied. Total RNA was subjected to mRNA enrichment using the NEBNext Poly(A) mRNA Magnetic Isolation Module (E7490, NEB), followed by the NEBNext Ultra II Directional RNA Library Prep Kit for Illumina (E7760, NEB) according to the manufacturer’s guidelines with minor adjustments. In brief, 1 μg total RNA was used to enrich the polyadenylated RNA fraction. The obtained RNA was fragmented for 12 min at 94 °C to target the insert size around 400 bp, followed by extended first- and second-strand synthesis. To enrich adaptor-ligated molecules, samples were pre-amplified with four cycles and left aside on ice. To determine an optimal number of polymerase chain reaction (PCR) cycles and limit the possibility of generating PCR duplicates and artifacts, a subsequent quantitative PCR reaction on previously pre-amplified samples was performed. Final libraries were validated in terms of quality and quantity by a Quantus fluorometer (Promega) and Tapestation 2020 High Sensitivity D1000 assay (Agilent Technologies), respectively. Pair-end sequencing (2 × 150 bp) was performed on an Illumina NovaSeq 6000 using a NovaSeq 6000 S4 flow cell (Illumina) to target a depth of 25–30 million reads per sample. The experimental setup involved three replicates per condition (*stim2* KO *vs*. control), consisting of 35 eyes per repetition.

The FastQC tool (v. 0.11.8) was used to assess quality of the obtained raw RNA-seq reads, followed by read mapping to the zebrafish reference genome (GRCz11 v. 109) using STAR aligner (v. 2.7)^81^, resulting in a mappability rate of 82–87%. Sequencing reads were further analyzed in the R programming language (v. 4.0.2), and DEGs were identified by the DESeq2 package^82^ according to the default workflow. Principal component analysis was performed on normalized read counts that were transformed to the log2 scale by the plotPCA function from the same package. The ggplot2 package was used for plot generation.

### Single-cell RNA-seq

#### Isolation of single-cell suspension

Larvae at 5 dpf were anesthetized by tricaine methanesulfonate MS222 (cat. no. E10521, Sigma-Adlrich) and heads were dissected in Dulbecco’s Modified Eagle Medium/Nutrient Mixture F-12 (cat. no 21041025, Gibco) with 10% fetal bovine serum (Gibco). Eyes were surgically removed from the zebrafish to focus on cells of neuronal origin. Single-cell suspensions were obtained by the enzymatic and mechanical digestion^41^ of heads without eyes that were isolated from two zebrafish lines (*stim2* KO and control).

#### Isolation of cells of neuronal origin

Immediately after the isolation of single-cell suspensions, the cells were sorted based on GCaMP5G fluorescence (488 nm excitation wavelength, 510 nm emission wavelength) to obtain cells of neuronal origin. GCaMP5 was expressed under the *elav/Huc* promoter, which is an early marker of neuronal cells^40,83^. Cell sorting was performed with a BD FACSAria II (BD Biosciences, Franklin Lakes, NJ, USA) with support from the Core Facility at IIMCB in Warsaw (Fig. 3S). Cell viability was measured using the trypan blue staining method. When the viability of cells was ~ 80%, the cells were immediately loaded on the 10× Chromium system for droplet encapsulation.

#### Droplet encapsulation, library preparation, and sequencing

Approximately 8000 cells were sorted and suspended in a ~ 40 μl volume. Cells were loaded according to the Chromium single cell 3′ kit’s standard protocol (V3 chemistry). To prepare the cells for droplet-based sequencing, GCaMP5G-positive single-cell suspensions were carefully mixed with reverse transcription mix before loading the cells on the 10X Genomics Chromium system according to the standard manufacturer’s protocol. During encapsulation, the cells were lysed within the droplet, and they released polyadenylated RNA bound to the barcoded bead, which was encapsulated with the cell. Following the 10X Genomics user manual’s guidelines, the droplets were directly subjected to reverse transcription, the emulsion was broken, and cDNA was purified using Silane beads. After the amplification of cDNA with 13 cycles, purification and quantification were performed. The 10× Genomics single-cell RNA-seq library preparation (involving fragmentation, dA-tailing, adapter ligation, and 12-cycle indexing PCR) was performed. After quantification, the libraries were sequenced at the Nencki Institute of Experimental Biology on an Illumina NextSeq 550 machine using a HighOutput flow cell in paired-end mode (R1: 26 cycles; I1: 8 cycles; R2: 57 cycles), thus generating 80–125 million fragments.

### scRNA-seq data analysis and identification of cell clusters

We obtained primary assembly of the zebrafish genome (*Danio rerio* GRCz11) and transcriptome annotation files from ensembl (release 105). Raw reads from fastq files were mapped to the aforementioned zebrafish reference genome, and gene expression was quantified with the STARSolo mode of STAR^84^ for each sample with the following parameters: --soloType CB_UMI_Simple, --soloCBwhitelist 3M-february-2018.txt (from Cell Ranger), --soloUMIlen 12, --soloCBmatchWLtype 1MM_multi_Nbase_pseudocounts, --soloUMIfiltering MultiGeneUMI_CR, --soloUMIdedup 1MM_CR, --soloCellFilter EmptyDrops_CR). In the preprocessing step that was conducted with the Seurat package (v. 5.0.1)^85^, we filtered out genes that were not expressed in at least three cells and kept only cells that expressed at least 100 genes and contained less than 10% mitochondrial genes. Additionally, to avoid doublets, cells with more than 2500 genes were removed. After this filtering step, 22889 cells were kept in both control and *stim2* KO samples with 20968 features identified. We then integrated all datasets from four samples (two replicates each for transgenic/control and *stim2* KO conditions) with the harmony package (v. 1.2)^86^.

The neighborhood graph was embedded using UMAP, and Leiden clustering (resolution = 1) was performed. The FindAllMarkers, FindConservedMarkers, and FindMarkers functions of Seurat were used to identify marker genes per cluster and differential gene expression in general between *stim2* KO and control for each cluster, as well as for all clusters per the entire dataset. We utilized curated gene expression data from the Daniocell database (daniocell.nichd.nih.gov) for all cell types in zebrafish neural tissues and eye neuronal cells and compiled a list of marker genes with the highly expressed genes in each cell type. The nomenclature used for the cell clusters is based on common gene markers associated with specific cell types. Consequently, some cluster names may appear to refer to retinal cell types, such as “retinal interneurons”, despite analysis of samples obtained from the brain. This phenomenon is attributable to the overlap of specific gene expression patterns between brain and retinal cell types, reflecting their shared developmental origins and functional similarities, thus these clusters represent brain cells expressing markers similar to those found in retinal cell types, rather than actual retinal cells.

The proportion of cells was calculated using the propeller function with arcsin square root transformation of proportions from the speckle v. 1.2 package^87^. Plots of proportions of cells were generated with GraphPad Prism software. Gene Ontology terms were identified with the topGO v. 2.54 package for DEGs or for marker genes in each cluster^88^. All steps that required the R programming language were performed in R v. 4.3.1.

### Gene Ontology

To identify functionally related genes the elim method from the topGO v. 2.54.0 and the org.Dr.eg.db v. 3.18.0 R packages was used^88^. Two gene sets were used to identified GO terms enrichment. First gene set were selected from marker genes for clusters (identified by the FindAllMarkers function from the Seurat package; *p*-value adjusted less than 0.05). Second gene set was selected from differentially expressed genes (DEGs) in each cluster (identified by the FindMarkers function from the Seurat package; *p*-value adjusted less than 0.05). In both cases gene identifiers with *p*-value adjusted greater than 0.05 (calculated with FindAllMarkers or FindMarkers functions from the Seurat package) were used as background genes. The statistical significance for GO terms enrichment was calculated with Fisher’s exact test.

### Transmission electron microscopy of zebrafish larvae

Zebrafish larvae at 5 dpf were euthanized using Tricane. The animals were fixed in 2.5% glutaraldehyde for 24 h at 4 °C, washed in phosphate-buffered saline (PBS), and postfixed with 1% osmium tetroxide for 1 h, followed by washing with water and staining with 1% aqueous uranyl acetate overnight at 4 °C. Next, the larvae were dehydrated with increasing concentrations of ethanol at room temperature and infiltrated with epoxy resin (Sigma Aldrich, St. Louis, MO, USA, catalog no. 45-359-1EA-F). Samples were then taken for polymerization for 48 h at 60 ºC. Polymerized blocks were trimmed with tissue processor (Leica EM TP) and cut with an ultramicrotome (EM UC7, Leica) to make ultrathin (70 nm thick) sections, which were collected on nickel grids (200 mesh, Agar Scientific, catalog no. G2200N). Specimen grids were examined with a Tecnai T12 BioTwin transmission electron microscope (FEI, Hillsboro, OR, USA) that was equipped with a 16 megapixel TemCam-F416 camera (TVIPS GmbH) in the Microscopy and Cytometry Facility at IIMCB in Warsaw.

### Electron microscopy analysis of retina layers

The retina is a very well-organized structure. It is characterized by subsequent layers of cells and their processes. This enables easy identification of particular layers. In our analysis, we measured widths of the IPL and GCL, and we quantified IPL dendrites and GCL neurons. Widths of the IPL and GCL were manually measured with ImageJ software on a single low-magnification (1200 ×) image where the entire surface of the layer was visible. The number of dendrites was assessed from images that were obtained at a larger magnification (4800 ×). Individual images were composed in CorelDRAW to show the surface visible in the low-magnification image. The image prepared in this way was exported to a tiff bitmap. We identified IPL dendrites as an object with a single membrane and eventually with mitochondria and without the signs of microtubule bundles and vesicles. These last two features enable us to differentiate them from axons also present in this layer. Neurons of GCL were assessed on the base of the number of nuclei present in this layer and using images taken at a magnification of 1200x. All measurements were performed manually in ImageJ software. The obtained data were analyzed by GraphPad Prism 9.0 software using an unpaired *t*-test with Welch’s correction. For each genetic variant, at least four samples were analyzed. To test the normality of the data, the Shapiro–Wilk test was performed.

For measurements of mitochondria in photoreceptors, the ratio of cristae area to the area of mitochondria was calculated using ImageJ software in the three pairs of control and *stim2* KO zebrafish according to a previous report^89^. Each pair contained at least 40 electron microscopy images of mitochondria. The total number of mitochondria that were analyzed was 190 in *stim2* KO zebrafish and 160 in controls. The data were analyzed using an unpaired *t*-test.

### Visualization of retinal cells and counting

Zebrafish larvae at 5 dpf were euthanized using Tricane, fixed in 4% paraformaldehyde overnight, incubated in 30% sucrose overnight, transferred to optimal cutting temperature compound in liquid nitrogen, and cut into 20 µm sections using a Leica cryostat. Sections were dried overnight, incubated for 5 min at room temperature in PBS/1%Tween/0.1% dimethylsulfoxide, hydrated in PBS for 10–15 min, permeabilized for 10 min at RT in PBS/0.2% Triton X-100, blocked in 5% bovine serum albumin with Triton-X100 for 30 min at room temperature in a humid chamber, incubated with primary antibodies in 5% bovine serum albumin (for photoreceptors: monoclonal anti-opsin, catalog no. O4886, Sigma-Aldrich; for amacrine cells: anti-GABA, catalog no. 0000177418, Sigma-Aldrich) at room temperature overnight in a humid chamber, rinsed, and incubated with secondary antibody (for photoreceptors: Alexa Fluor 488 donkey anti-mouse IgG [H + L], lot no. 2147618, Invitrogen; for amacrine cells: Alexa Fluor 594 goat anti-rabbit IgG [H + L], lot no. WH322204, Invitrogen) in 5% bovine serum albumin at room temperature for 2 h in the dark in a humid chamber. Samples were washed and incubated for 15 min with Hoechst 33342 (Thermo Fisher; 1:1000 in PBS) at room temperature. After drying, mounting medium (Prolong Gold, catalog no. P36930, Invitrogen) was added to the slides. Z-stacks of images were acquired by confocal microscopy (Zeiss LSM 800; 40 × /1.3 oil objective).

To calculate cell densities of GABA-positive cells in the INL and opsin-positive cells in the outer nuclear layer, we applied a segmentation workflow in Imaris, Cellpose^90^, and ImageJ software. First, surfaces for both layers were drawn manually on three-dimensional image stacks in Imaris and converted to masks. Second, Hoechst-stained nuclei in the retina were three-dimensionally segmented using the “denoise” image restoration and cyto3 models in Cellpose^90^. Third, nuclear labels were thresholded for fluorescence signal, masked for each layer, thresholded for volume, and counted in ImageJ. To obtain cell densities, the counted nuclei were divided by mask volume. Because *stim2* KO retinas had substantially lower GABA signals than controls, the threshold for GABA fluorescence in the above workflow was calculated automatically with the “Adjust Threshold” tool in ImageJ (default setting) to retain GABA-positive structures that were comparable in shape and volume.

## Supplementary Information

Supplementary Information 1.

Supplementary Information 2.

Supplementary Information 3.

Supplementary Information 4.

Supplementary Information 5.

## Data Availability

All data generated or analyzed during this study are included in this article (and its ementary Information files). RNA sequencing data (bulkRNAseq, scRNAseq) are available in the GEO repository under the accession number GSE264312 .
