# Supplementary material for: Loss of Stim2 in zebrafish induces glaucoma-like phenotype
Source: Sci Rep. 2024 Oct 18;14:24442. doi: 10.1038/s41598-024-74909-0 (PMC11489432; doi:10.1038/s41598-024-74909-0)
Supplement: Supplementary file 1 — Supplementary Information 1. [file 41598_2024_74909_MOESM1_ESM.docx]

**Supplementary Data**


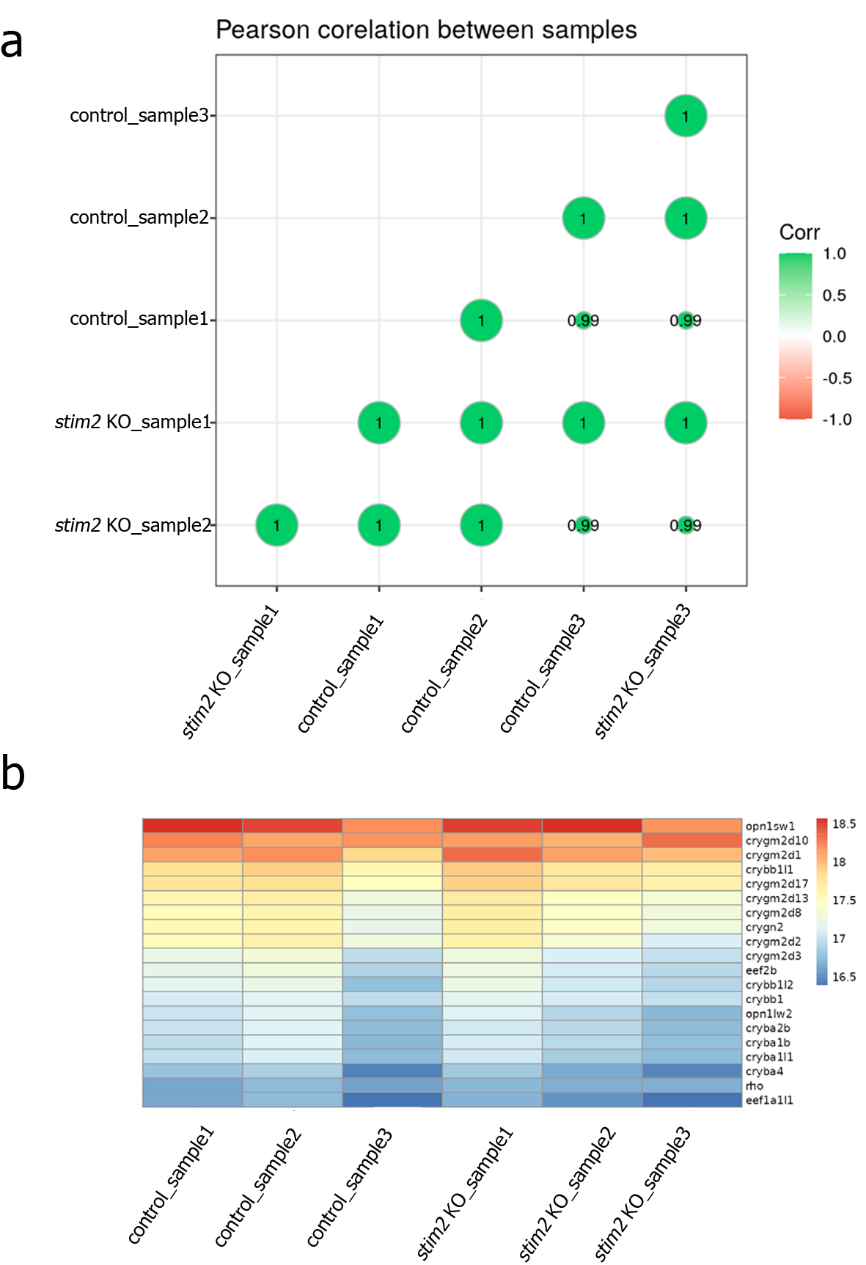


Fig. 1S. Expression of genes in eyes of *stim2* KO and control zebrafish identified by RNA-seq. (a) Correogram of high similarity between tested conditions. All samples were highly similar to each other. (b) Top 20 most expressed genes based on log-normalized count data among tested conditions, confirming specificity of the eye origin.


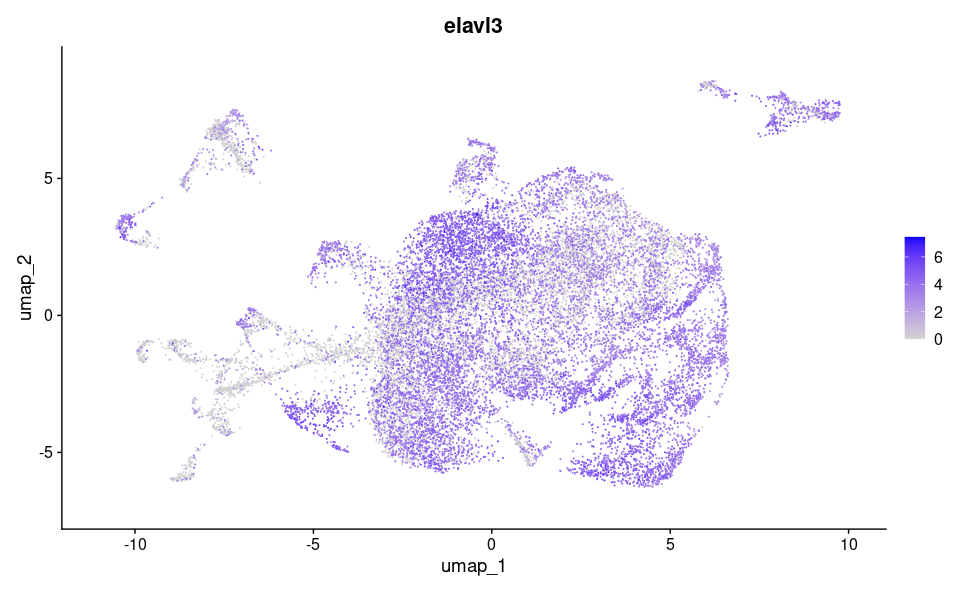


Fig. 2S. Uniform Manifold Approximation and Projection (UMAP) visualization of single-cell RNA sequencing data showing the expression of the neuronal marker gene *elavl3* across brain cells of 5 days post-fertilization (dpf) zebrafish larvae. Each point represents an individual cell, with the position determined by UMAP dimensions 1 and 2, which are reflecting the major axes of variation in gene expression profiles among cells. The color intensity indicates the expression level of *elavl3*, with darker blue representing higher expression and lighter shades indicating lower expression.


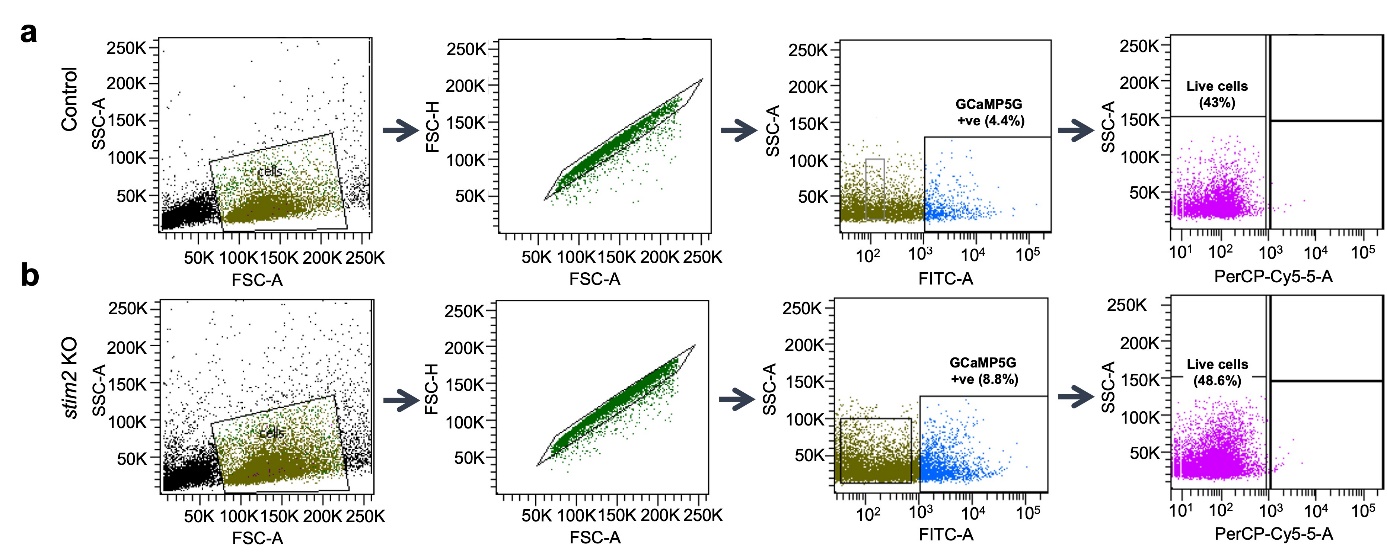


Fig. 3S. Cells were sorted by fluorescence-activated cell sorting (FACS) based on their green fluorescence to select neuronal cells. (a) Control group and (b) *stim2* KO zebrafish. The first scatter plot in each panel shows the initial gating strategy to select cells based on forward scatter (FSC-A) and side scatter (SSC-A) characteristics. The second plot shows the singlet cell gate using FSC-A and FSC-H. The third plot shows the selection of GCaMP5G-positive cells based on FITC-A fluorescence, indicating neuronal cells, with the percentage of GCaMP5G-positive cells. The final plot shows live/dead cell discrimination using PerCP-Cy5-5-A fluorescence, with the percentage of live cells indicated. The control group has 4.4% GCaMP5G-positive cells and 43% live cells, while the stim2 KO group has 8.8% GCaMP5G-positive cells and 48.6% live cells.


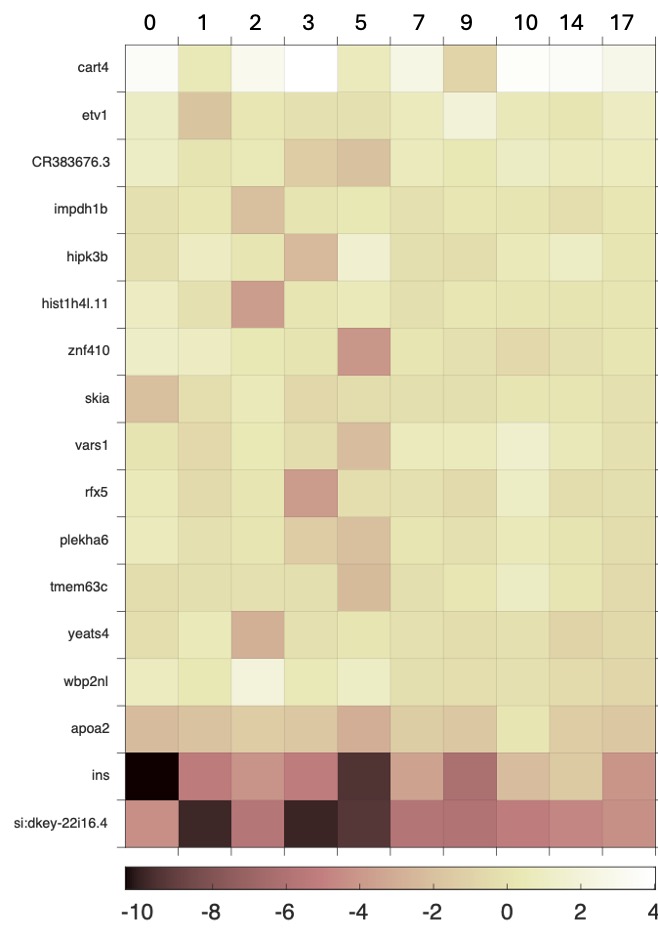


Fig. S4. The heatmap illustrates the differential expression of various genes in brain cells of 5 days post-fertilization (dpf) zebrafish larvae. Each row represents a different gene, while each column corresponds to a specific cell cluster with DEGs. The color intensity represents the log-fold change in gene expression, with the color scale ranging from -10 (dark red, indicating downregulation) to 4 (light yellow, indicating upregulation).

Table 1S. Top genes expressed in unassigned clusters in zebrafish brain cells of neuronal origin. BP, biological process; CC, cellular component; MF, molecular function.

| **Cluster number** | **Top genes characteristic (markers) of clusters (according to “FindAllMarkers” function of Seurat package)** | **Top GO terms calculated for characteristic genes (markers) for clusters** |
| --- | --- | --- |
| *O^Δ^* | *zgc:158463*, *cnbpb*, *nucks1a*, *csnk1a1*, *celf2*, *aplp1*, *anp32a*, *cirbpa*, *rab10*, *C19H6orf62* | GO:0006397: mRNA processing (BP)  GO:0000122: negative regulation of transcription by RNA polymerase II (BP)  GO:0030097: hemopoiesis (BP)  GO:0007178: transmembrane receptor protein serine/threonine kinase signaling (BP)  GO:0000785: chromatin (CC)  GO:0140513: nuclear protein-containing complex (CC)  GO:1990904: ribonucleoprotein complex (CC)  GO:0005634: nucleus (CC)  GO:0005515: protein binding (MF)  GO:0003682: chromatin binding (MF)  GO:0003729: mRNA binding (MF)  GO:0003723: RNA binding (MF) |
| *6^Δ^* | *neurod1*, *fat2*, *zbtb18*, *neurod6b*, *neurod2*, *eomesa*, *bhlhe22*, *bhlhe23*, *zic2a*, *draxin* | GO:0061564: axon development (BP)  GO:0006412: translation (BP)  GO:0031018: endocrine pancreas development (BP)  GO:0070925: organelle assembly (BP)  GO:0022625: cytosolic large ribosomal subunit (CC)  GO:1902495: transmembrane transporter complex (CC)  GO:0022627: cytosolic small ribosomal subunit (CC)  GO:0000275: mitochondrial proton-transporting ATP synthase complex, catalytic sector F(1) (CC)  GO:0070888: E-box binding (MF)  GO:0003735: structural constituent of ribosome (MF)  GO:0003723: RNA binding (MF)  GO:1901363: heterocyclic compound binding (MF) |
| *11^Δ^* | *tmsb*, *rpl19*, *rps20*, *hsp90ab1*, *h3f3d*, *rpl24*, *rps19*, *rpl28*, *rplp1*, *rpl17* | GO:0006412: translation (BP)  GO:0043009: chordate embryonic development (BP)  GO:0000028: ribosomal small subunit assembly (BP)  GO:0002181: cytoplasmic translation (BP)  GO:0022625: cytosolic large ribosomal subunit (CC)  GO:0022627: cytosolic small ribosomal subunit (CC)  GO:0032040: small-subunit processome (CC)  GO:0005840: ribosome (CC)  GO:0003735: structural constituent of ribosome (MF)  GO:0019843: rRNA binding (MF)  GO:0070180: large ribosomal subunit rRNA binding (MF)  GO:0005198: structural molecule activity (MF) |
| *24^Δ^* | *si:dkey-117i10.1*, *kiss1*, *gpr139*, *csrp2*, *si:dkeyp-72h1.1*, *prox1a*, *prkcq*, *plekhg5b*, *gpr78b*, *wnt11* | GO:0007186: G protein-coupled receptor signaling pathway (BP)  GO:0007417: central nervous system development (BP)  GO:0007346: regulation of mitotic cell cycle (BP)  GO:0048884: neuromast development (BP)  GO:0030425: dendrite (CC)  GO:0005615: extracellular space (CC)  GO:0008076: voltage-gated potassium channel complex (CC)  GO:0071944: cell periphery (CC)  GO:0005249: voltage-gated potassium channel activity (MF)  GO:0061134: peptidase regulator activity (MF)  GO:0005179: hormone activity (MF)  GO:0004930: G protein-coupled receptor activity (MF) |
